# *N*-Confused Porphyrin Immobilized on Solid Supports: Synthesis and Metal Ions Sensing Efficacy

**DOI:** 10.3390/molecules23040867

**Published:** 2018-04-10

**Authors:** Sara R. D. Gamelas, Ana T. P. C. Gomes, Nuno M. M. Moura, Maria A. F. Faustino, José A. S. Cavaleiro, Carlos Lodeiro, Marta I. S. Veríssimo, Tiago Fernandes, Ana L. Daniel-da-Silva, M. Teresa S. R. Gomes, Maria G. P. M. S. Neves

**Affiliations:** 1 Department of Chemistry and QOPNA, University of Aveiro, 3810-193 Aveiro, Portugal; sara.gamelas@ua.pt (S.R.D.G.); jcavaleiro@ua.pt (J.A.S.C.); 2 BIOSCOPE Group, LAQV@REQUIMTE, Chemistry Department, Faculty of Science and Technology, University NOVA of Lisbon, 2829-516 Caparica, Portugal; cle@fct.unl.pt; 3 ProteoMass Scientific Society, Madan Park, Rua dos Inventores, 2825-182 Caparica, Portugal; 4 Department of Chemistry and CESAM, University of Aveiro, 3810-193 Aveiro, Portugal; mverissimo@ua.pt (M.I.S.V.); mtgomes@ua.pt (M.T.S.R.G.); 5 Department of Chemistry and CICECO, University of Aveiro, 3810-193 Aveiro, Portugal; jtfernandes@ua.pt (T.F.); ana.luisa@ua.pt (A.L.D.-d.-S.)

**Keywords:** *N*-confused porphyrins, organic/inorganic hybrids, metal cations, aqueous solution

## Abstract

In this work, the *N*-confused porphyrin 5,10,15,20-tetraphenyl-2-aza-21-carbaporphyrin (NCTPP) was immobilized on neutral or cationic supports based on silica and on Merrifield resin. The new materials were characterized by appropriate techniques (UV-Vis spectroscopy, SEM, and zeta potential analysis). Piezoelectric quartz crystal gold electrodes were coated with the different hybrids and their ability to interact with heavy metals was evaluated. The preliminary results obtained showed that the new materials can be explored for metal cations detection and the modification of the material surface is a key factor in tuning the metal selectivity.

## 1. Introduction

The effects associated with overpopulation and pollution is putting at risk an essential element on earth for living beings—WATER. In particular, wastewaters from industrial, agricultural, and domestic activities are commonly discharged without adequate treatment into the water bodies, contributing to an increase in water pollution [1,2,3,4]. Among the pollutants commonly found (e.g., dyes, organic solvents, and chlorine compounds) heavy metals merit special attention [5]. The detection and removal of trace amounts of heavy metal ions, e.g., Tl(I), Hg(II), and Cd(II) are of particular mindful owing to their high toxicity for living organisms and due to their non-biodegradability and bioaccumulation with relevant noisome consequences on the environment and human health. In fact, the heavy metal ions can accumulate in human internal organs and induce disorders such as renal and central nervous system dysfunction or even cancer. When in the environment, these pollutants can reach the freshwater supplies making them unfit for human consumption [6,7,8,9,10]. Also, the detection of metal cations, such as Zn(II), Cu(II), or Cr(III), with relevant functions on biological and environmental fields merit to be studied [11,12,13,14,15,16,17]. Therefore, the development of new and more efficient probes for detection and determination of trace amounts of heavy and transition metal ions is a subject of great concern.

Nowadays, several methods are used for monitorization, detection, and removal of toxic heavy metal cations, such as resin chelation [18], ion exchange [19], membrane filtration [20], coprecipitation [21], and liquid-liquid extraction [22]. Nevertheless, these techniques show several disadvantages, namely, the use of high amounts of solvents, a non-selective elimination of the target heavy metal ions, and laborious and expensive sample handling. So, it is essential to develop efficient alternatives able to detect and to remove heavy metal ions with higher sensitivity and selectivity.

The analytical approaches that can be used to detected and quantify heavy metals in water are flame atomic absorption spectrometry, atomic, visible, and fluorescence spectrometry, cathodic adsorptive stripping voltammetry, anodic stripping voltammetry, and electrochemical impedance spectroscopy (EIS) which often requires extensive sample preparation [7,23]. Alternatively, coating the surface of a piezoelectric quartz crystal with some complexing structures allows detecting heavy metal ions in aqueous solutions in real time by monitoring the frequency of oscillation [23]. The frequency of oscillation of a piezoelectric quartz crystal decreases with the mass deposited on its active area [24].

Porphyrins and analogues, well known by their impressive applications in several areas such as catalysis [25,26,27,28], photocatalysis [29,30] electronic materials [31,32,33], supramolecular chemistry [34,35], biomimetic models [36,37] , and medicine [25,38,39,40,41,42,43], are also often used as sensor probes due to their unique photophysical and photochemical properties [25,35,44,45,46]. In fact, porphyrins as free-bases or as metallo complexes have been used to create the so-called artificial noses and tongues for gas, cations, anions, and neutral species detection [47]. Commonly, porphyrinic macrocycles functionalized in the *meso*-positions with hydrogen bond donors (e.g., amine or amide) allow an extensive modulation of their anionic guest recognition [47,48,49,50]. Alternatively, the presence of a Lewis acidic environment in the porphyrinic core (usually Zn(II)) enables anion binding. In general, metal ion porphyrin-based probes include *meso* functionalized porphyrins with crown ether, amines, amides, or pyridines moieties, showing sensing ability towards metal ions as Zn(II), Hg(II), Cd(II), or Pb(II) [51]. In the last years, our group is giving also a significant contribution to the development of *beta*-functionalized porphyrins for the detection of heavy metal ions [9,52,53,54].

The efficacy of *N*-confused porphyrins (NCP) (a porphyrin isomer in which one of the pyrrole rings is connected to *meso*-carbons at α and β′ positions) [55] is also meriting some attention as sensor probes; these inverted porphyrins show unique binding properties for halide anions via hydrogen bond formation or deprotonation at the peripheral *N*-H [56,57]. In addition to outer *N*-H moiety, the axial positions of metal centers in *N*-confused porphyrin complexes could also be used as the anion-binding site [58]. Although, the interesting features exhibited by NCP, a literature survey shows that there is a scarce number of studies involving NCPs for heavy metal ion probes [46].

So, in this work, we envisage that the immobilization of 5,10,15,20-tetraphenyl-2-aza-21-carbaporphyrin (NCTPP) through its peripheral *N*-H on neutral or cationic supports based on 3-bromopropyl-functionalized silica (Si) and on Merrifield resin (MF) could afford new materials with excellent features to coat piezoelectric quartz crystal electrodes in order to follow their interaction with heavy metals in a real-time monitoring. For simplicity and time-saving, as the purpose of this study was to compare the metal complexation ability of the immobilized NPC with a cationic non-immobilized NCTPP bearing a propyl group (NCTPP-Pr^+^), experiments were not followed until equilibrium but were performed by flow injection methodology under careful flow control.

## 2. Results and Discussion

### 2.1. Synthesis and Immobilization of NCTPP

**Figure 1:**
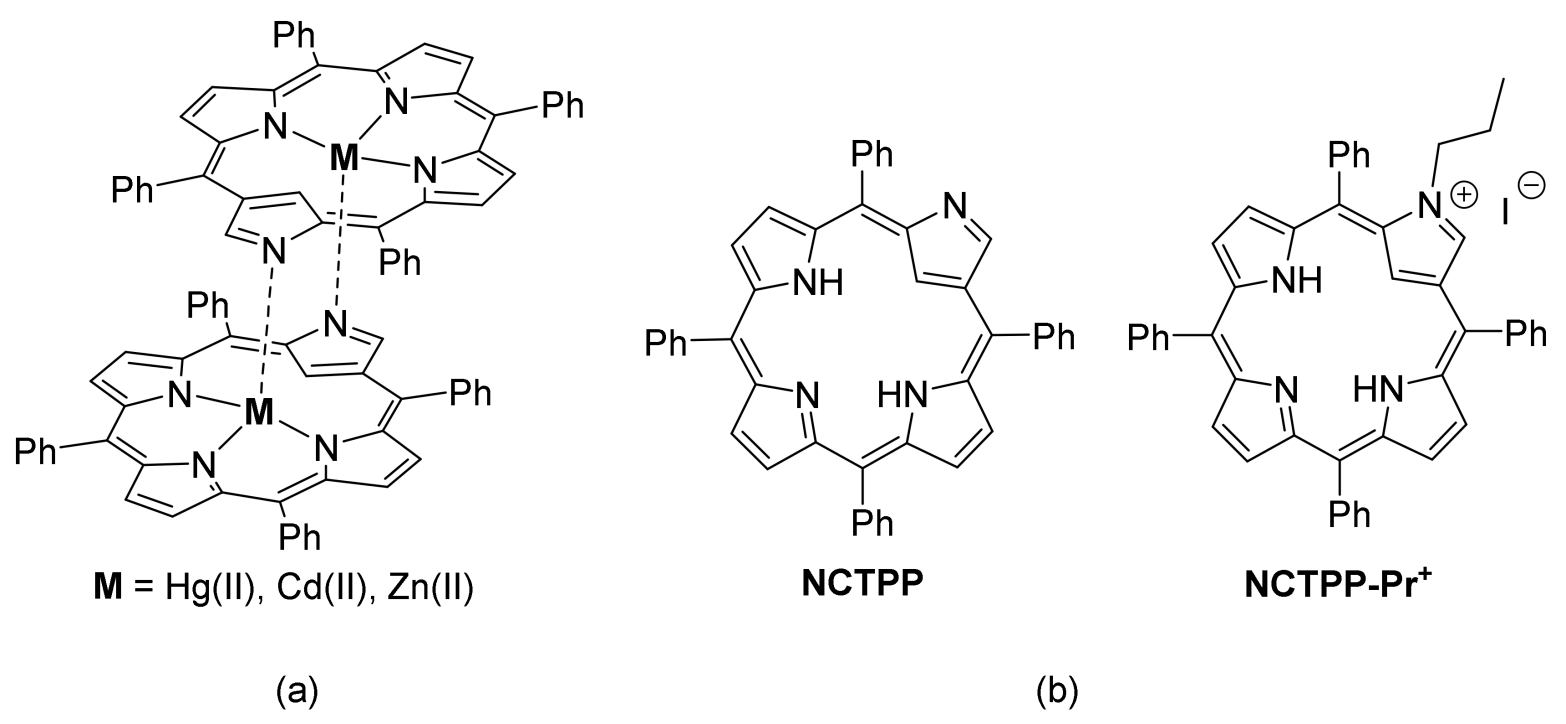
(**a**) Sandwich type complex of *N*-confused porphyrins and (**b**) Structures of NCTPP and NCTPP-Pr^+^ derivatives.

NCTPP was prepared following the Lindsey methodology as reported in the literature [59]. *N*-confused porphyrins can complex with a wide range of metals, (e.g., Zn(II), Cu(II), Ag(III), Pd(II), Cd(II), and Hg(II)) into the macrocycle core and in the peripheral inverted nitrogen, leading in some cases to sandwich type complexes (Figure 1a) [60,61,62]. However, when the peripheral inverted nitrogen is protected, e.g., by alkylation, it loses its Lewis acid behaviour and only the nitrogens into the inner core are able to interact with metal cation ions [63,64].

In this study, in order to avoid the formation of a sandwich type complex, thereby assuring a similar geometry in immobilized NCTPP and non-immobilized NCTPP and to ensure that the inner core nitrogens are the unique site able to interact with heavy metal cations, the alkylation of NCTPP was performed using propyl iodide, according to a literature procedure, affording the expected NCTPP-Pr^+^ (Figure 1) [65].

**Scheme 1:**
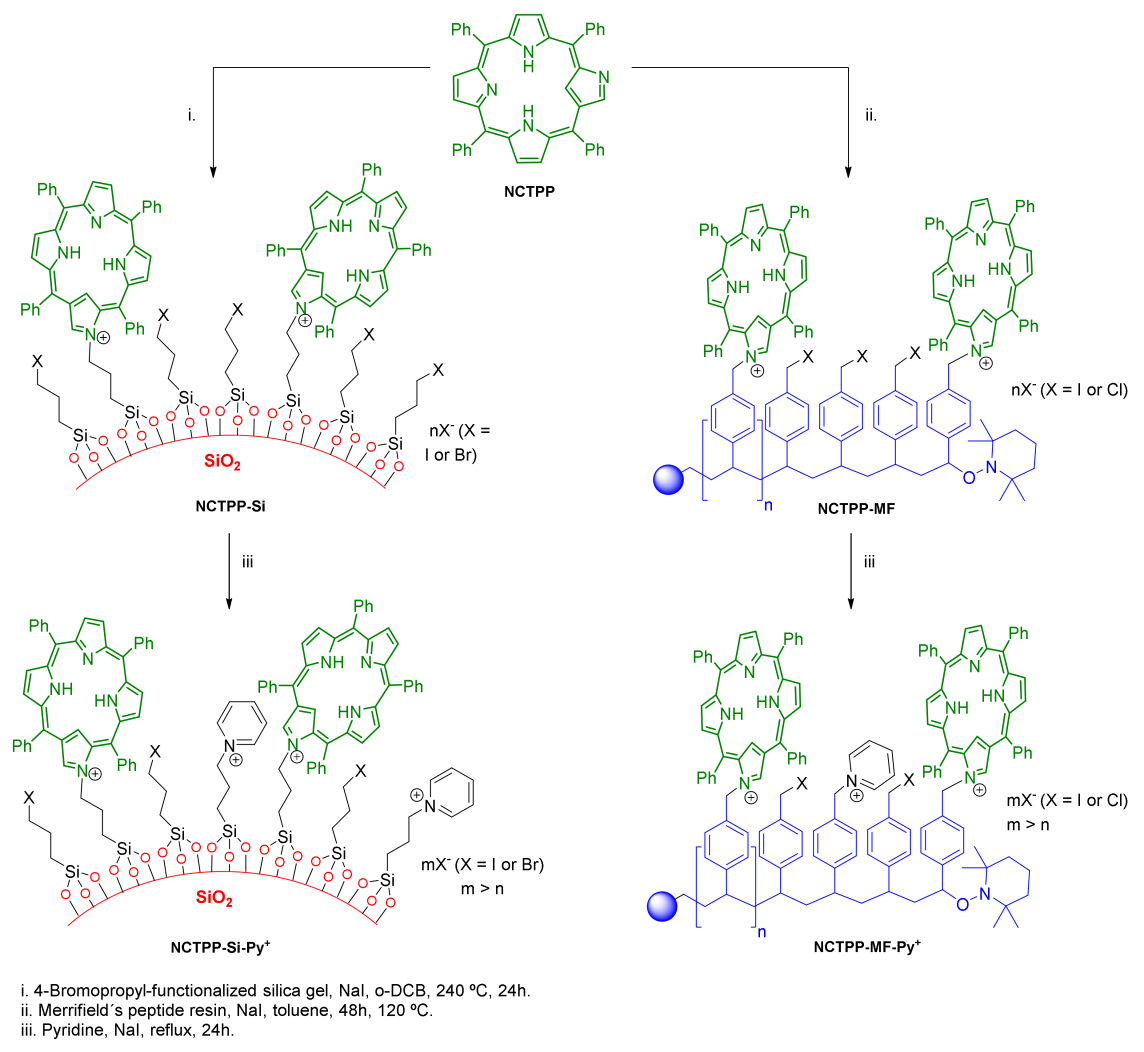
Immobilization of NCTPP on a functionalized silica gel (Si) and on a Merrifield resin (MF).

The immobilization of NCTPP in 3-bromopropyl functionalized silica (Si) and in Merrifield peptide resin (MF) was performed according to the experimental procedure summarized in Scheme 1 [66]. The reactions were carried out in the presence of NaI, under reflux in the adequate solvent (*o*-DCB for Si, and toluene for MF) until the total consumption of the starting *N*-confused porphyrin was observed by thin layer chromatography (TLC) [66]. The desired materials NCTPP-Si and NCTPP-MF were obtained with a load of 2.0% and 8.0% (*w*/*w*) of NCTPP, respectively.

**Scheme 2:**
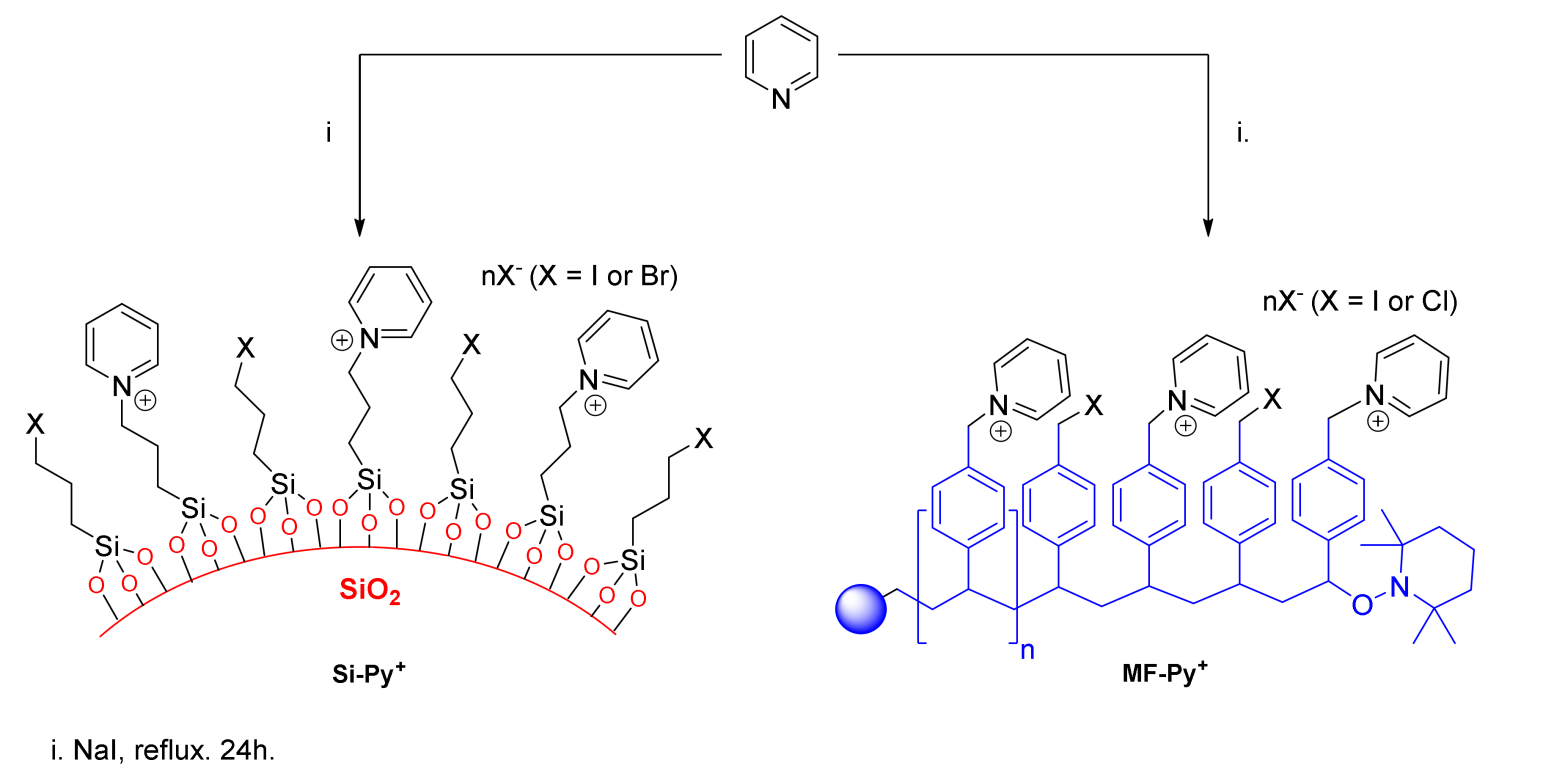
Cationization of the materials Si and MF with pyridine.

To study how the sensing ability of the novel materials would be affected by extra cationization, NCTPP-Si and NCTPP-MF were treated with pyridine. In a typical experiment, each material was heated in pyridine and in the presence of NaI. After 24 h the reaction mixtures were filtered, washed and dried affording the new materials labelled as NCTPP-Si-Py^+^ and NCTPP-MF-Py^+^ (Scheme 1). Also, the solid supports Si and MF were submitted to pyridine treatment using similar experimental conditions affording Si-Py^+^ and MF-Py^+^, respectively (Scheme 2).

### 2.2. Photophysical and Morphological Properties

#### 2.2.1. UV-Vis Spectroscopy

**Figure 2:**
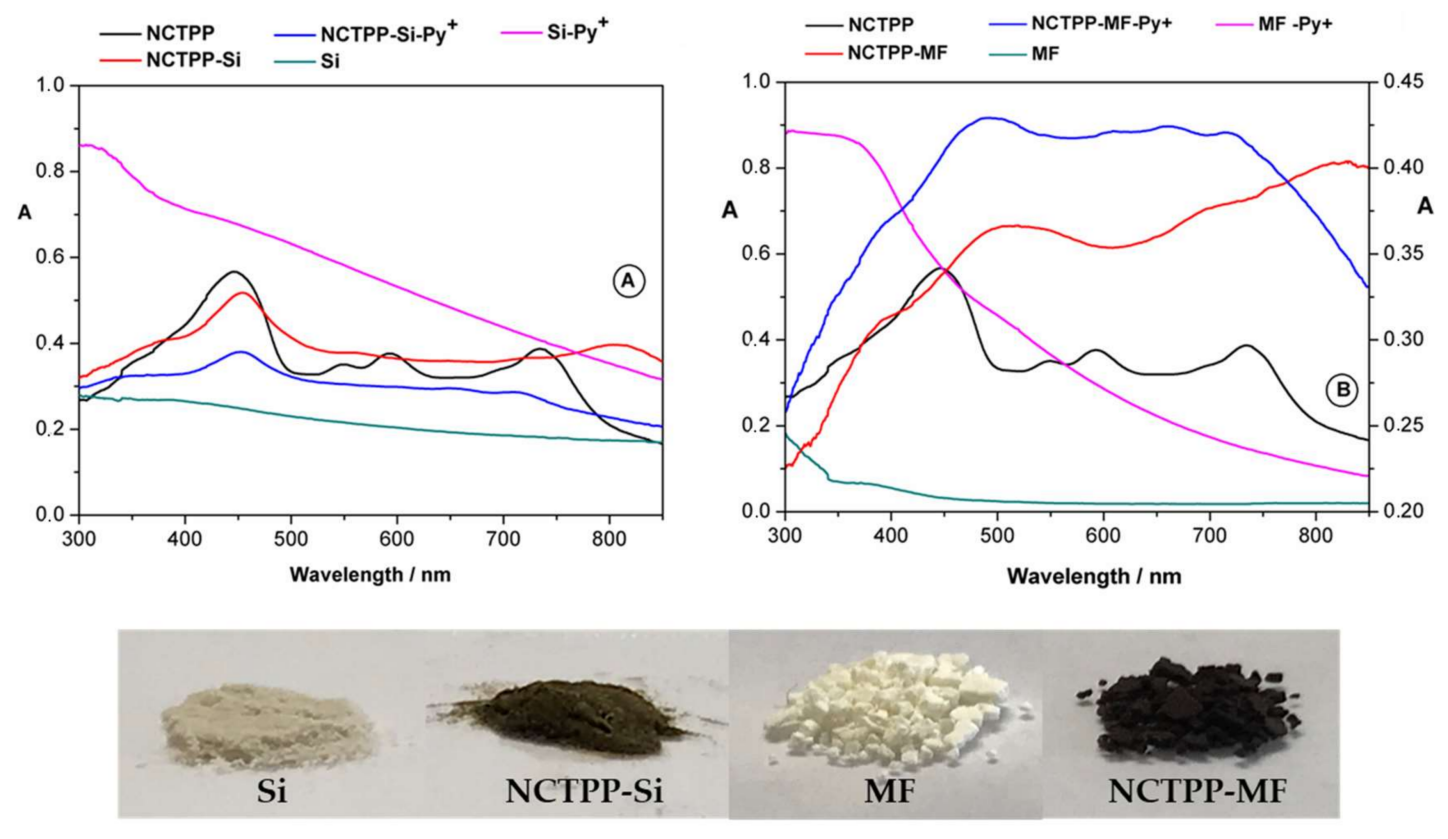
Top: Solid state UV-Vis spectra of (**A**) NCTPP, Si, and organic/inorganic-hybrids and (**B**) NCTPP, MF, and organic/inorganic hybrids. Bottom: visual appearance of Si and MF materials before and after NCTPP immobilization.

The solid-state absorption spectra presented in Figure 2 evidence the success of the NCTPP immobilization process, as well as the extra functionalization with pyridine moieties.

The solid-state absorption spectrum of NCTPP evidences the typical feature of a free base *meso*-tetraaryl *N*-confused porphyrin due to π–π* transitions, with a Soret band centred at 449 nm and three Q bands between 550 and 739 nm (Figure 2). Considering the absorption spectra of the materials based on silica, NCTPP-Si and NCTPP-Si-Py^+^, in both cases the Soret band suffered a red-shift of ca. 8 nm. In the case of the NCTPP-Si, the last Q band suffered also a red-shift of ca. 70 nm, while the correspondent band of NCTPP-Si-Py^+^ is blue-shifted 25 nm (Figure 2A). The bathochromic shift observed for NCTPP-Si and NCTPP-Si-Py^+^ can be attributed to the porphyrinic core distortion.

The absorption spectra of NCTPP-MF and NCTPP-MF-Py^+^ do not show well-defined absorptions bands from the *N*-confused porphyrinic moiety. However, it is clearly observed the presence of a hyperchromic effect in the absorbance region from 400 to 800 nm of both derivatives, due to the presence of the tetrapyrrolic macrocycle (Figure 2B). The broadening observed in these spectra seem to indicate that during the immobilization process a significant distortion in porphyrin conformation occurred, which can be related with alterations in the aromatic arrangement, affecting the porphyrin ring symmetry.

As expected the Si and MF supports do not show absorption in the UV-Vis region, however after being submitted to cationization with pyridine, the spectra of Si-Py^+^ and MF-Py^+^ presents an absorption enhancement in the ultraviolet region with a new band at *ca*. 350 nm; this feature is due to the presence of the pyridinium moieties.

#### 2.2.2. SEM

**Figure 3:**
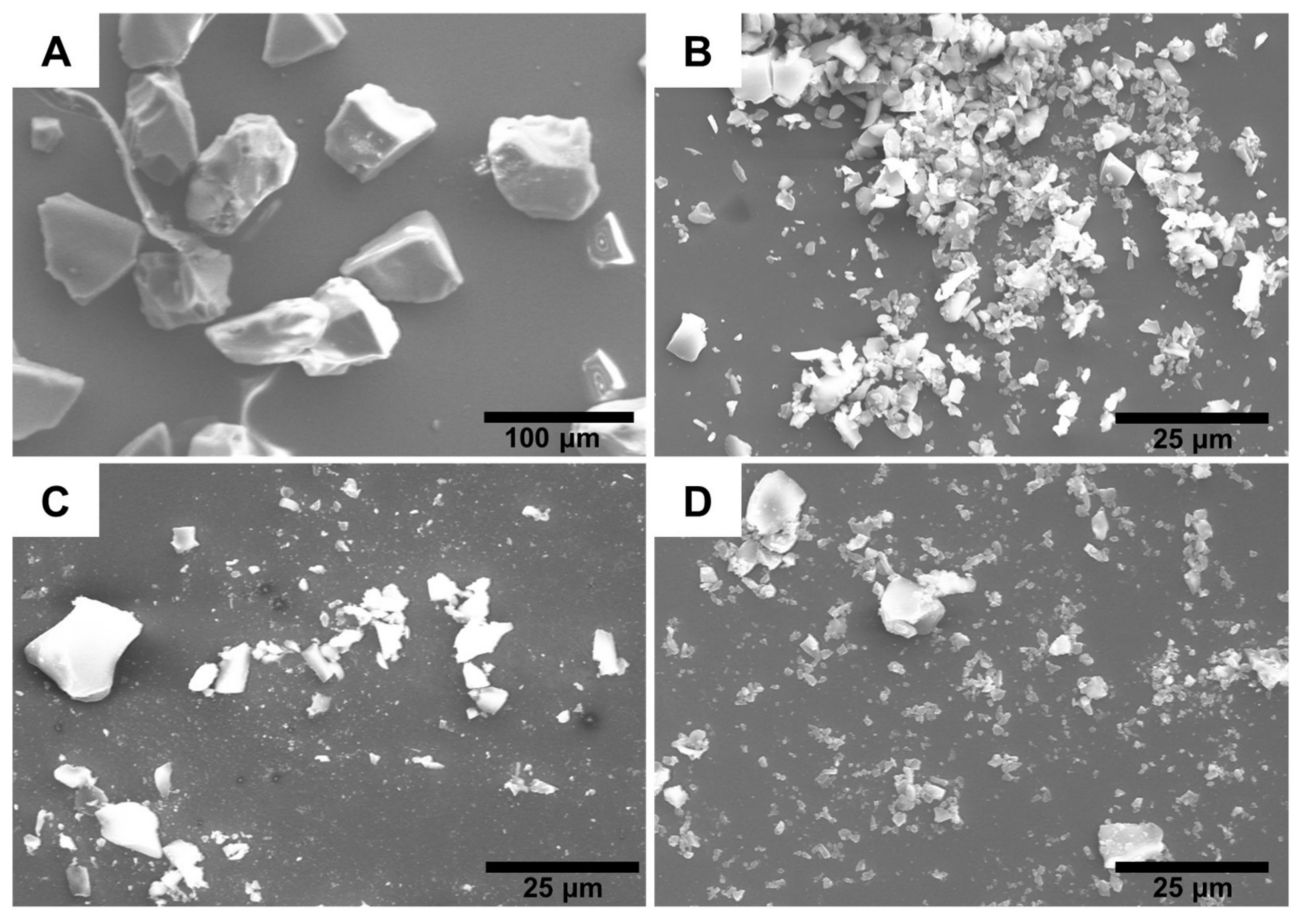
SEM images of (**A**) Si, (**B**) Si-Py^+^, (**C**) NCTPP-Si and (**D**) NCTPP-Si-Py^+^.

**Figure 4:**
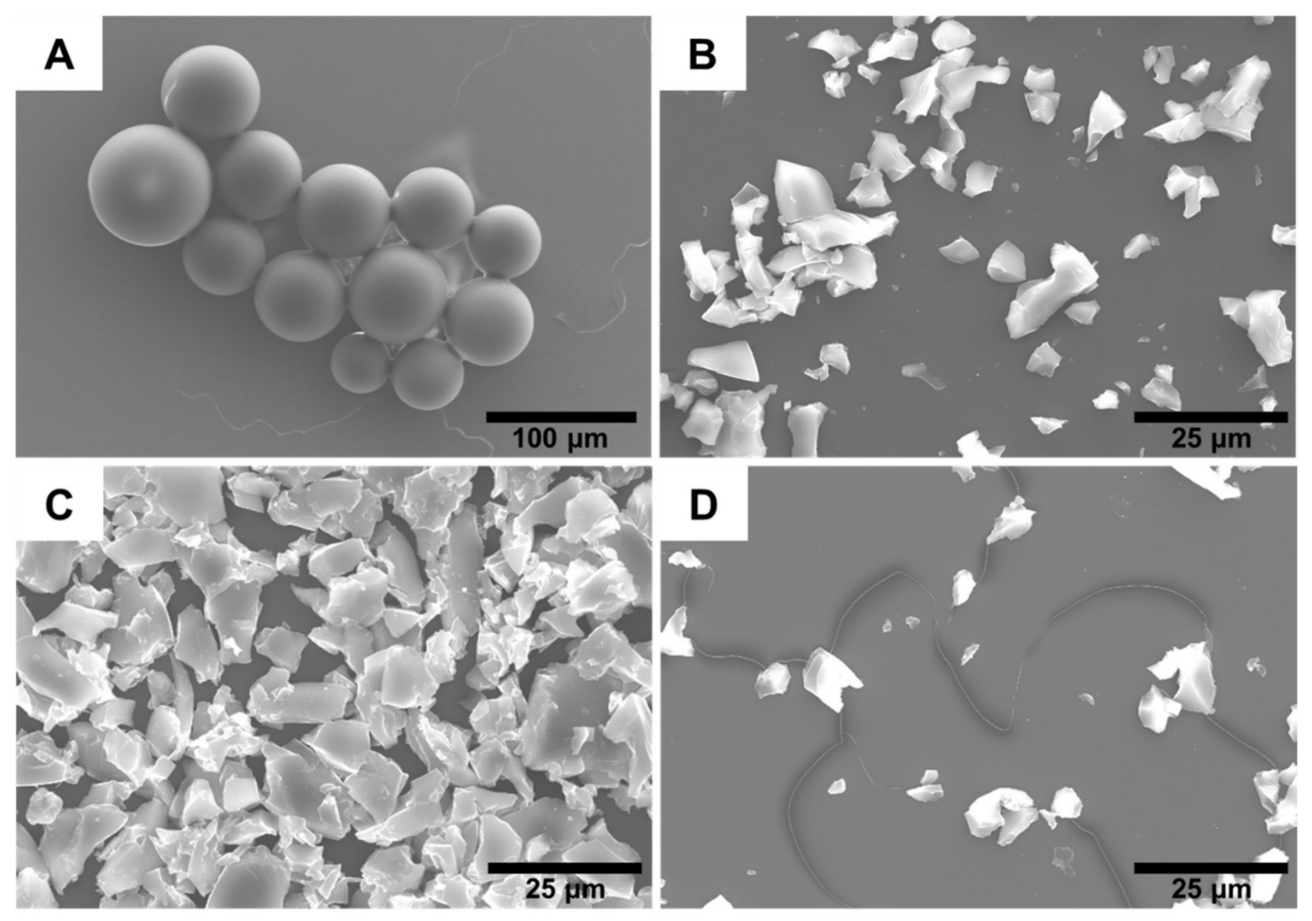
SEM images of (**A**) MF, (**B**) MF-Py^+^, (**C**) NCTPP-MF, and (**D**) NCTPP-MF-Py^+^.

The morphological characteristics of the solid support Si, MF and of the respective porphyrinic hybrids were analysed by scanning electron microscopy (SEM). The Si support appeared as particles with irregular shape and dimensions between 20 and 75 µm. The particle size decreased to below 18 µm either after the immobilization of the NCTPP or after being submitted to pyridine treatment. The hybrid NCTPP-Si-Py^+^ comprised irregular shaped particles with polydisperse size distribution from 500 nm to 6 µm and larger particles with dimensions nearly 12 µm (Figure 3). The beads of MF were spherical and uniform and presented irregular shape after the immobilization of NCTPP and pyridine treatment. Likewise, hybrid NCTPP-MF-Py^+^ particles were irregular in shape, with particle size ranging from 3 to 12 µm (Figure 4).

### 2.3. Zeta Potential

**Figure 5:**
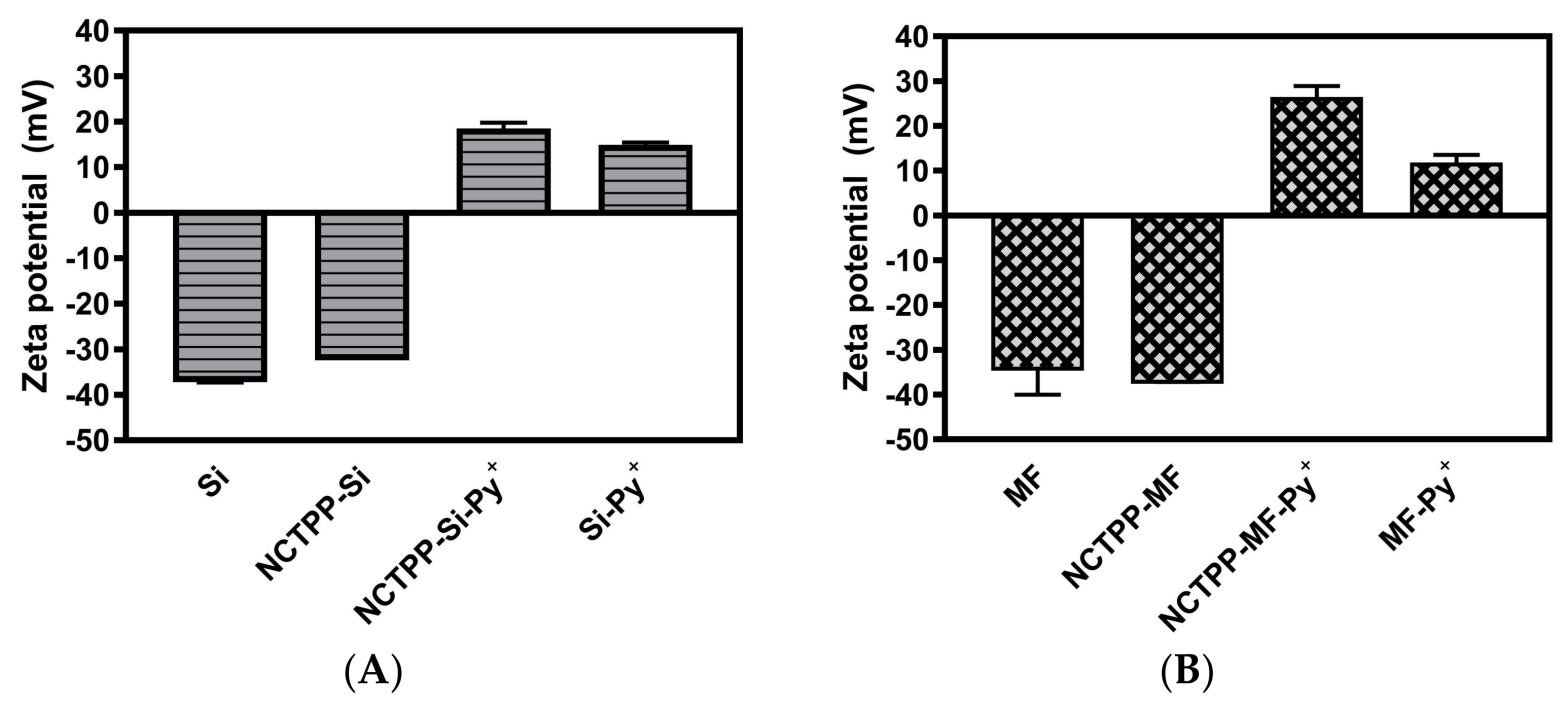
Zeta potential values of Si (**A**) and MF (**B**) and the corresponding organic-inorganic hybrids.

The surface charge of the materials was assessed by zeta potential measurements in aqueous suspensions at a pH ranging from 5.2 to 5.6 (Figure 5A,B). At this pH the surface of 3-bromopropyl functionalized silica (Si) and Merrifield resin (MF) are highly negatively charged (ζ = −35.9 ± 1.1 mV and ζ = −33.7 ± 5.1 mV, respectively) and no significant changes of the zeta potential were observed after the immobilization of the NCTPP on its surface (NCTPP-Si: ζ = −31.1 ± 0.5 mV and NCTPP-MF: ζ = −36.7 ± 0.3 mV). The Br^-^ anions in Si and the Cl^-^ anions in MF are electronegative elements which confers a negative density and charge to the surface of the materials. The amount of NCTPP which was immobilized was not enough to change the superficial charge of the materials. As expected, the treatment with pyridine of the silica-based hybrid resulted in the reverse of the surface charge of the material to positive values (NCTPP-Si-Py^+^: ζ = 18.5 ± 0.3 mV, Figure 5A). Identical behaviour was found in the silica submitted to similar pyridine treatment (Si-Py^+^, Figure 5A). In the Merrifield -based materials, the cationization with pyridine yielded materials also with positively charged surface NCTPP-MF-Py^+^ (ζ = 26.5 ± 2.0 mV) and MF-Py^+^ (ζ = 9.1 ± 1.6 mV) (Figure 5B).

### 2.4. Metal-Receptor Interaction

In order to test the ability of NCTPP-Pr^+^, NCTPP-Si, NCTPP-Si-Py^+^, NCTPP-MF, and NCTPP-MF-Py^+^ to interact with the heavy metals Tl(I) and Cr(III) in aqueous solutions, piezoelectric quartz crystal gold electrodes were coated with the different compounds and its frequency shifts were recorded after interaction with chosen metals. Quartz crystals were coated by depositing a drop of a solution of the selected porphyrin on the surface and allowing it to dry. NCTPP-Pr^+^ was dissolved in THF, while the NCTPP immobilized in silica or in the Merrifield resin were mixed with polyvinyl chloride (PVC) and a plasticizer 2-nitrophenyl octyl ether (NPOE), in 1:33:69 (*w*/*w*) proportion and suspended in THF. The piezoelectric quartz crystal used in the present work possessed gold electrodes, which do not interact with the target metals. Therefore, coating the piezoelectric crystal with a sensitive layer with affinity to those metals is essential to detect them, and it is a main task in sensor preparation.

Tests for the recognition capability and sensitivity were done by injecting solutions of the two different metals (Tl(I) and Cr(III)) into a water stream. This water stream was continuously flowing through the sensor cell at a rate of 0.75 mL min^−1^. When the metal, carried by the water flow, reached the sensor, its interaction with the coating induced a decrease in the frequency of oscillation of the piezoelectric quartz crystal. As water continues to flow, it sweeps the metal away, and frequency recovers its baseline value in less than 2 min, showing the reversibility of the metal-coating interaction, compatible with a weak attachment, very far from the establishment of a covalent bonding. 

**Figure 6:**
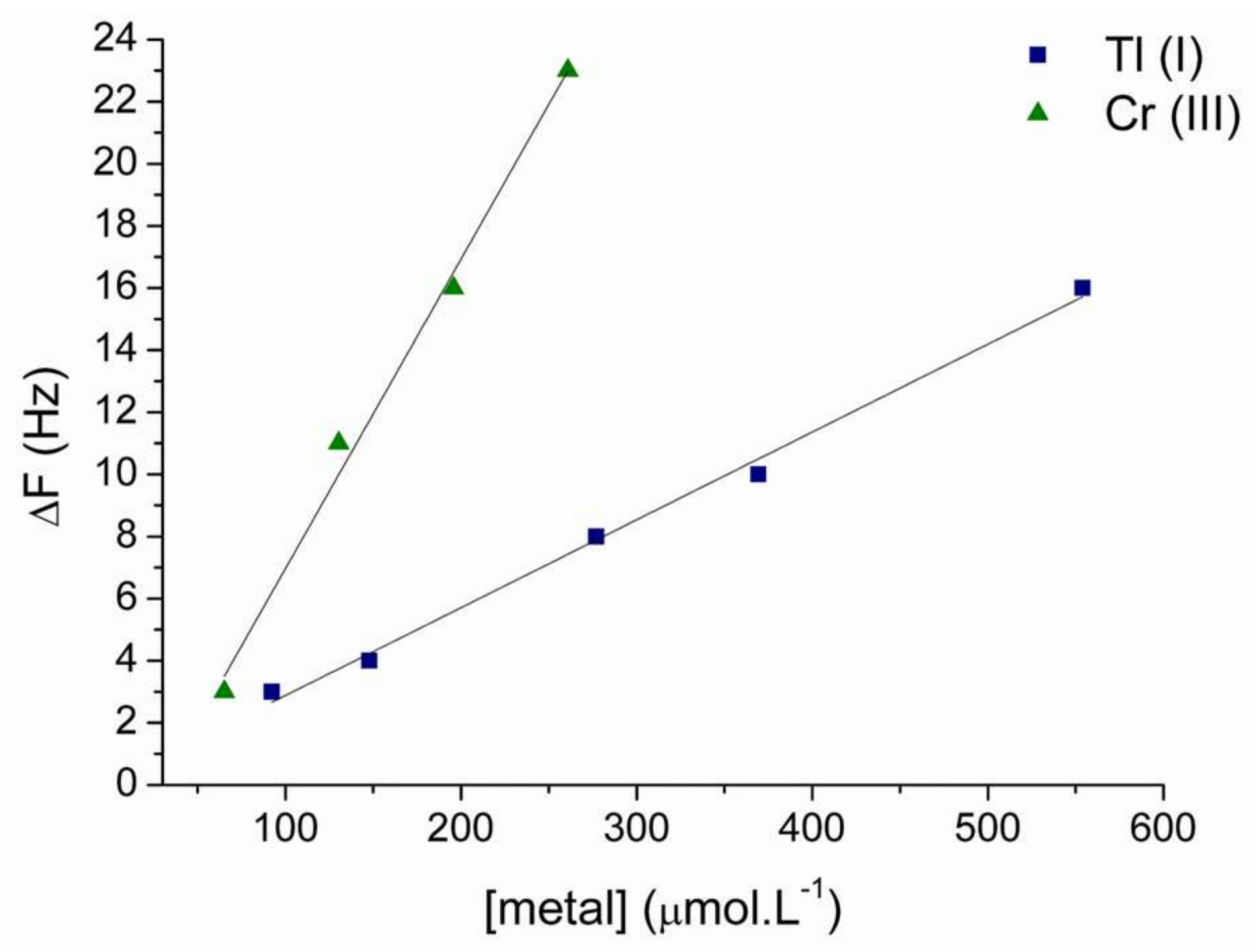
Response of the sensor based on NCTPP-Pr^+^ to: Tl^+^ (blue) and Cr^3+^ (green).

**Figure 7:**
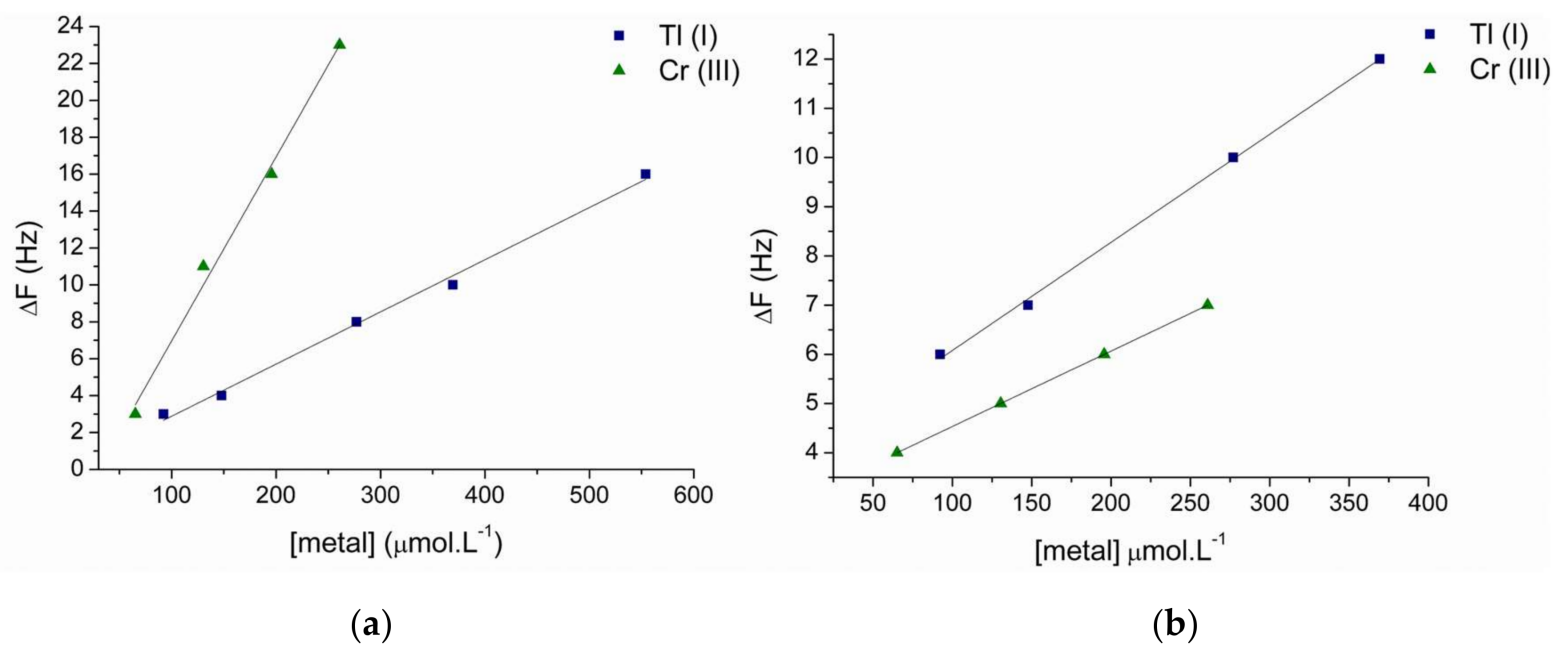
Response of the sensor to Tl^+^ (blue) and Cr^3+^ (green) based on: (**a**) NCTPP-Si and (**b**) NCTPP-Si-Py^+^.

**Figure 8:**
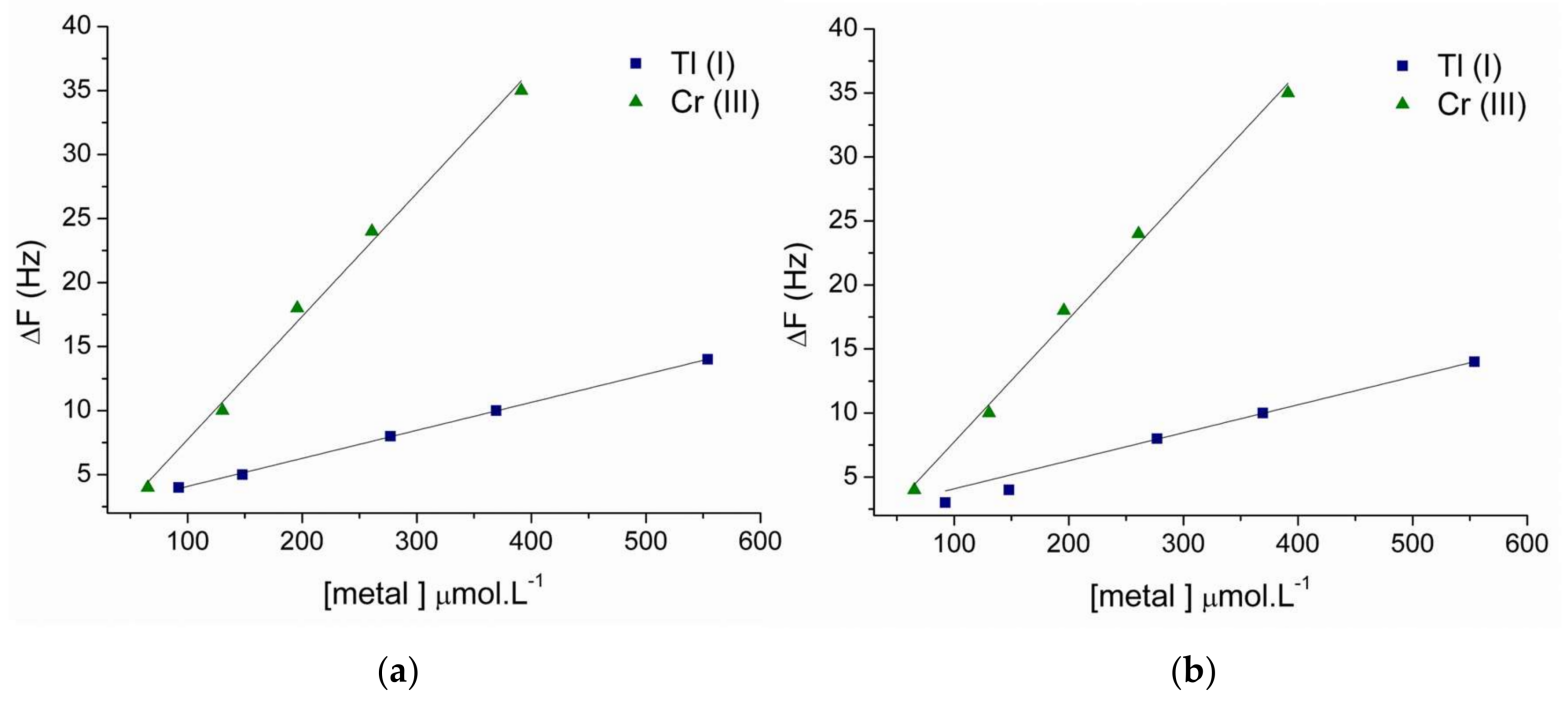
Response of the sensor to Tl^+^ (blue) and Cr^3+^ (green) based on: (**a**) NCTPP-MF and (**b**) NCTPP-MF-Py^+^.

The linearity of the frequency versus the concentration was checked far from the coating saturation [67]. Figure 6, Figure 7 and Figure 8 show the responses of the sensors to the metal solutions. Based on the obtained responses, sensitivity was calculated relatively to each metal for each material and the results are displayed in Table 1.

According to the results, all sensors, independently of the porphyrin being free or immobilized on Si or on MF, responded to thallium and chromium (Figure 6, Figure 7 and Figure 8). Four of the five materials show higher sensitivity to chromium regarding thallium which was not expected, attaining to the gravimetric response of the sensors. The atomic weight of thallium is around four times the atomic weight of chromium. Therefore, according to mass, it is expected, to have a higher response for thallium ions comparing with an equal number of chromium ions. The response of the sensor for solutions with the same ionic concentration was surprisingly higher for chromium. Cr^3+^ ionic radius is smaller than Tl^+^ ionic radius, which can contribute to enhancing the density of ions able to interact with the porphyrin molecules. For the sensor coated with NCTPP-Pr^+^ the slope of the calibration line for Cr^3+^ is 3.52 times the slope of the calibration line for Tl^+^, but this ratio increases to 4.39 for the sensor of the porphyrin supported on MF resin. This increase may be related to the negative zeta potential of the material, and the oxidation number of chromium, which is +3, therefore higher than the oxidation number of thallium, which is +1. This significant advantage on the sensitivity to Cr^3+^ over Tl^+^ of the porphyrin supported on the MF membrane is lost (slope for Cr^3+^ is 2.29 times the slope for Tl^+1^) with the pyridine introduction, as it turns the material positively charged. 

After supporting the porphyrin on Si, sensitivity to chromium diminishes in relation to sensitivity to thallium, even with negative zeta potentials. Comparing with the porphyrin supported on the Merrifield membrane, the magnitude of zeta potential is lower and the density of porphyrin on the solid support is also lower. The decrease in the steric hindrance effect would allow the larger thallium ions to approach the porphyrin molecules. Not surprisingly, the introduction of the pyridinium units and the resultant positive net charge is another negative factor affecting chromium sensitivity, rendering this material more sensitive to thallium than to chromium.

## 3. Materials and Methods 

### 3.1. General Remarks

The ^1^H-NMR was recorded in a Bruker AMX 300 NMR spectrometer or Bruker AMX 700 NMR (Wissembourg, France) at 300.13 MHz and 700.13 MHz, respectively, where CDCl_3_ was used as the solvent and TMS was used as internal reference. The chemical shifts are expressed in δ (ppm) and the coupling constants (*J*) in Hertz (Hz). Mass spectra were recorded using MALDI TOF/TOF 4800 Analyzer, Applied Biosystems MDS Sciex (Sciex, Darmstadt, Germany), with CHCl_3_ as solvent and without matrix. The UV-Vis spectra were recorded on a UV-2501PC Shimadzu spectrophotometer (Shimadzu, Kyoto, Japan) using CHCl_3_ as solvent. For the diffuse reflectance characterization of the solids (diluted with MgO) was used a UV-Vis Jasco V560 spectrophotometer (JASCO International Co., Ltd., Tokyo, Japan). 

Analytical TLC was carried out on precoated sheets with silica gel (Merck 60, 0.2 mm thick, Darmstadt, Germany). Column chromatography was carried out using silica gel (Merck, 35–70 mesh). Preparative thin-layer chromatography was carried out on 20 × 20 cm glass plates coated with silica gel (0.5 mm thick).

All reagents used in this work were purchased from Sigma Aldrich (Steinheim, Germany) and not subject to any purification process, being directly used in the reactions due to its elevated purity. Solvents were used as received or distilled and dried by using standard procedures according to the literature [68].

3-Bromopropyl-functionalized silica gel (200–400 mesh; extent of labelling: 1.5 mmol g^−1^ loading) and Merrifield’s resin (100–200 mesh, extent of labelling: 3.5–4.5 mmol g^−1^ Cl^−^ loading, 1% cross-linked) were also purchased from Sigma-Aldrich.

5,10,15,20-tetraphenyl-2-aza-21-carbaporphyrin (NCTPP) and 2-*N*-propyl-5,10,15,20-tetraphenyl-2-aza-21-carbaporpyrin (NCTPP-Pr^+^) were prepared according with literature procedures. The characterization of NCTPP and NCTPP-Pr^+^ were performed by ^1^H-NMR, UV-Vis and mass spectrometry with all the experimental data being in agreement with the described literature data [59,65].

NCTPP: ^1^H-NMR (300 MHz, CDCl_3_): δ −5.10 (1H, s, inner-CH), −2.51 (2H, s, *N*H), 7.74–7.79 (9H, m, H-*m*,*p*-Ph-5,10,15), 7.81-7.86 (3H, m, H-*m*,*p*-Ph-20), 8.14–8.19 (4H, m, H-*o*-Ph-10,15), 8.32–8.38 (4H, m, H-*o*-Ph-5,20), 8.40–8.46 (3H, m, β-H), 8.49 (1H, d, *J* = 5.0 Hz, β-H), 8.63 (1H, s, β-H-3), 8.80 (1H, d, *J* = 4.9 Hz, β-H), 8.86 (1H, d, *J* = 4.9 Hz, β-H) ppm. UV-Vis (DMF): λ_max_ (log ε) 451 (5.08), 554 (3.95), 599 (3.75), 698 (4.07) 737 (4.02) nm. MS [MALDI (+)]: *m*/*z* 615 [M + H]^+^.

NCTPP-Pr^+^: ^1^H-NMR (700 MHz, CDCl_3_): δ 0.41 (3H, t, *J* = 7.3, CH_3_), 1.22–1.35 (2H, m, CH_2_), 3.57 (2H, t, *J* = 7.6 Hz, *N*-CH_2_), 7.46 (1H, d, *J* = 4.4 Hz, β-H-8), 7.50 (1H, d, *J* = 4.2 Hz, β-H-17), 7.54–7.57 (6H, m, H-*m*,*p*-Ph-10,15), 7.62–7.64 (6H, m, H-*m*,*p*-Ph-5,20), 7.71–7.74 (2H, m, β-H-12,13), 7.82 (1H, d, *J* = 4.4 Hz, β-H-7), 7.83–7.84 (4H, m, H-*o*-Ph-10,15), 7.92 (1H, d, *J* = 4.2 Hz, β-H-18), 7.95–7.98 (4H, m, H-*o*-Ph-15,20), 8.02 (1H, s, β-H-3) ppm. UV-Vis (DMF): λ_max_ (log ε) 445 (4.57), 550 (3.29), 600 (3.58), 652 (3.90) 706 (4.04) nm. MS [MALDI (+)]: *m*/*z* 657 [M]^+^.

### 3.2. NCTPP Immobilization on Functionalized Silica (Si) and on Merrifield Resin (MF)

#### 3.2.1. Synthesis of NCTPP-Si

In a sealed tube was added 500 mg of 3-bromopropyl functionalized silica (Si), *meso*-tetraphenyl-2-aza-21-carbaporphyrin, NCTPP, (10 mg, 0.0163 mmol), sodium iodide (20.0 mg, 0.133 mmol) and *o*-DCB (5.0 mL). The mixture was stirred for 24 h at 240 °C. After cooling, the resulting brown solid was filtered and washed successively using CH_2_Cl_2_, CH_2_Cl_2_/CH_3_OH (9:1), CH_3_OH, acetone and distilled water. After washing, the new material was dried overnight in the oven. The total immobilization of NCTPP in the functionalized silica was calculated considering the difference between the amount initially used and the one recovered in the washing solvents (determined by UV-Vis). The prepared NCTPP-Si was obtained with 2% (*w*/*w*) of immobilized NCTPP.

#### 3.2.2. Synthesis of NCTPP-MF

In a round-bottom flask, a mixture containing 250 mg of Merrifield´s peptide resin (MF), NCTPP, (20.0 mg, 0.0326 mmol), and NaI (20.0 mg, 0.133 mmol) in dry toluene (5 mL) was maintained under stirring for 48 h at 120 °C. After this time, the crude mixture was cooled down, filtered and the resulting solid was washed using CH_2_Cl_2_, CH_2_Cl_2_/CH_3_OH (9:1), CH_3_OH, acetone and distilled water. After washing, the new material was dried overnight in the oven. The total immobilization of NCTPP in the functionalized silica was calculated by subtracting to the amount initially used the one recovered in the washing solvents (measured by UV-Vis). The prepared material NCTPP-MF was obtained with 8% (*w*/*w*) of immobilized NCTPP.

#### 3.2.3. Synthesis of NCTPP-Si-Py^+^ and NCTPP-MF-Py^+^

The appropriate NCTPP-Si (160 mg) or NCTPP-MF (150 mg) was treated with NaI (20.0 mg, 0.133 mmol) in pyridine (2.0 mL) and stirred for 24 h under reflux. The resulting mixtures were cooled, filtered and the respective solids were washed using CH_2_Cl_2_, CH_2_Cl_2_/CH_3_OH (9:1), CH_3_OH, acetone and distilled water. The solids were dried overnight in the oven yielding the adequate materials NCTPP-Si-Py^+^ and NCTPP-MF-Py^+^. 

#### 3.2.4. Si and MF Cationization

Materials Si and MF (without NCTPP) were also treated with pyridine under the same experimental conditions used above in order to obtain the correspondent positively charged surface Si-Py^+^ and MF-Py^+^.

### 3.3. Photophysical and Morphological Properties

#### SEM

For sample preparation for scanning electron microscopy (SEM) analysis, an aliquot of a dilute particle suspension in ethanol was allowed to air dry on glass slides and then was coated with evaporated carbon. SEM was performed using a scanning electron microscope Hitachi SU-70 operating at an accelerating voltage of 20 kV.

### 3.4. Zeta Potential

The surface charge of the nanoparticles was assessed in a folded capillary cell by zeta potential measurements performed in aqueous solutions of the particles (pH within the range 5.2 to 5.6), using Zetasizer Nano ZS equipment from Malvern Instruments (Malvern, UK).

### 3.5. Sensing Ability Towards Metal Cations

Acoustic wave sensors were prepared by depositing each one of the prepared materials on one of the faces of a piezoelectric quartz crystal. The coated quartz crystal was housed in a flow cell. The cell inlet was connected through a 1/16′′ tube to a manual injection valve with a 0.5 mL loop, while its output was connected to waste. A constant flow of 0.75 mL min^−1^ of distilled water was flowing over the coated piezoelectric crystal, assuring a stable baseline frequency of oscillation. The injection of a solution containing the metal ion at a known concentration able to interact with the coating produces a decrease in the oscillation frequency, which is proportional to the increase in the coating mass, produced by the metal. Neglecting possible differences in the acoustic properties of the coating and any changes promoted by the metal, observed frequency decreases depend on the coating amount, but for the same coating, magnitude of shifts is proportional to the molar amounts of metal physically or chemically linked to the coating, and to the atomic mass of the metal. The high sensitivity of these sensors, commonly known as quartz crystal microbalances, turns them into a superior technique to evaluate the affinity of each compound to specific metals.

## 4. Conclusions

In summary, novel organic/inorganic hybrids were successfully prepared by immobilization of 5,10,15,20-tetraphenyl-21-aza-carbaporphyrin on functionalized silica and Merrifield resin via a simple heterogeneous procedure, and the material surface was fully characterized by UV-Vis, SEM, and zeta potential techniques.

Piezoelectric quartz crystals with gold electrodes coated with these organic/inorganic hybrids were prepared and their sensing ability towards different metal ions was successfully explored and studied. Results showed that immobilized *N*-confused porphyrin retained their ability to complex with metals, which shows their potential use in metal cations remediation. Moreover, the interaction is reversible, and functionalized surfaces are easily recovered. Besides, the inclusion of charged groups allows tailoring the selectivity of the functionalized surfaces towards metallic cations.

Finally, this study proved to be a good preliminary assay of the use of these materials as sensitive coatings for real-time metal analysis in water. 

## Figures and Tables

**Table 1:** Summary of all the sensors sensitivity for each metal.

Material	Metal	Sensitivity (10^3^ Hz L mol^−1^)
NCTPP-Pr^+^	Tl (I)	28.3
	Cr(III)	99.7
NCTPP-Si	Tl (I)	35.4
	Cr(III)	98.9
NCTPP-Si-Py^+^	Tl (I)	21.9
	Cr(III)	15.0
NCTPP-MF	Tl (I)	21.9
	Cr(III)	96.1
NCTPP-MF-Py^+^	Tl (I)	26.3
	Cr(III)	60.1
